# Cholangiocarcinoma: a guide for the nonspecialist

**DOI:** 10.2147/IJGM.S186854

**Published:** 2018-12-20

**Authors:** Munirah Alsaleh, Zoe Leftley, Thomas A Barbera, Paiboon Sithithaworn, Narong Khuntikeo, Watcharin Loilome, Puangrat Yongvanit, I Jane Cox, Nittaya Chamodol, Richard RA Syms, Ross H Andrews, Simon D Taylor-Robinson

**Affiliations:** 1 Division of Surgery and Cancer, Imperial College London, London W2 INY, UK, s.taylor-robinson@imperial.ac.uk; 2 Cholangiocarcinoma Research Centre, Faculty of Medicine, Khon Kaen University, Khon Kaen 40002, Thailand; 3 Faculty of Life Sciences & Medicine, King’s College London, London SE5 9NT, UK; 4 Department of Electrical and Electronic Engineering, Imperial College London, London SW7 2AZ, UK

**Keywords:** cholangiocarcinoma, etiology, diagnosis, treatment, bile ducts

## Abstract

Cholangiocarcinoma (CCA) is a tumor with increasing prevalence around the world. The prevalence of CCA is highest in East Asia and most significantly in the countries through which the Mekong River flows, owing to the presence of liver flukes, which are consumed in raw fish dishes. Outside Asia, the causes of bile duct cancers for the most part are unknown. In this review, we assess the current state of knowledge in both fluke-associated and sporadic CCA, from etiological, diagnostic, and treatment perspectives.

## Introduction

**Figure 1:**
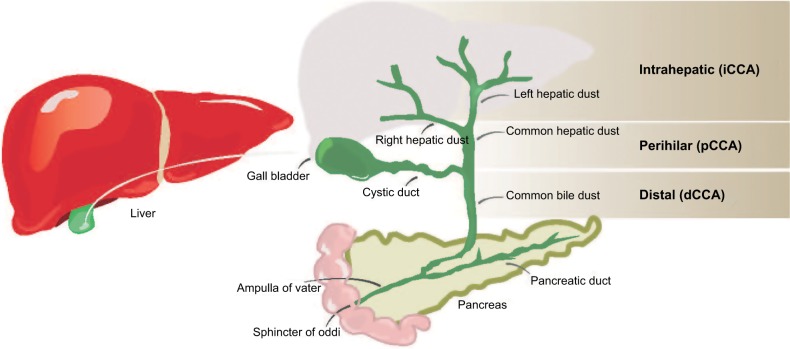
Locations of CCA in the biliary tree. **Note:** iCCAs arise within the hepatic biliary ducts, perihilar in the bile duct bifurcation, and distal tumors arise anywhere along the common biliary duct outside the liver. **Abbreviations:** CCA, cholangiocarcinoma; iCCA, intrahepatic CCA.

Cholangiocarcinoma (CCA), or bile duct carcinoma, is a malignancy that originates from the cholangiocytes lining the biliary tree. It is the second most common primary liver malignancy, following hepatocellular carcinoma (HCC), accounting for 10%–20% of all hepatic cancers.^1^ Based on where the tumor arises in the biliary tree, CCA tumors are classified into intrahepatic (iCCA) or distal, while tumors developing in the bile duct bifurcation are classified as perihilar (pCCA; Figure 1). The majority of CCA tumors are in the perihilar and distal regions, and only 10% are intrahepatic.^2^ The typical age at presentation is the seventh decade, with a slight male predominance.^3^

### Epidemiology

**Figure 2:**
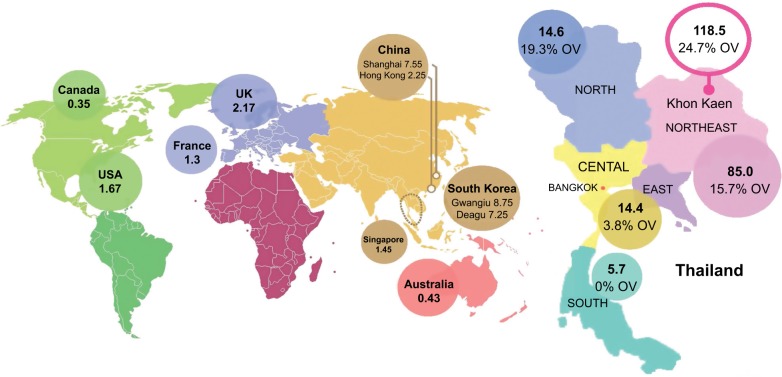
Estimated global CCA incidence. **Notes:** World map showing CCA incidence per 100,000 (left), and incidence of CCA and prevalence of *Opisthorchis viverrini* in Thailand from 1990 to 2001 (right). %OV=*Opisthorchis viverrini* prevalence. Adapted from Bragazzi M, Cardinale V, Carpino G. Cholangiocarcinoma: epidemiology and risk factors. *Transl Gastrointest Cancer*. 2012;1(1):21–32 with permission from AME Publishing Company^6^ (left) and Data from Keiser and Utzinger^7^ (right). **Abbreviation:** CCA, cholangiocarcinoma.

Recent studies have shown an unexplained global increase in CCA incidence and mortality. For instance, in England, the number of CCA deaths increased from 1,720 in 2010 to 2,161 in 2013.^4^ A similar trend was found in the United States; the incidence rate of iCCA increased by 165% in 30 years, from the1970s to the 1990s, from 0.32 per 100,000 to 0.85 per 100,000.^1^ The geographical distribution of CCA cases is uneven (Figure 2), and it closely mirrors the prevalence of its predisposing risk factors. The vast majority of CCA cases in the Western developed nations have a sporadic nature. Primary sclerosing cholangitis (PSC), a chronic condition causing biliary obliterative fibrosis, is the commonest risk factor in Western countries, but it is associated with only 10% of cases, indicating the diversity of influencing factors on disease risk.^5^ The highest reported CCA incidence internationally is in Northeast of Thailand, 118.5 per 100,000, in Khon Kaen, which is some 100 times higher than the global rate. The high CCA burden is associated with liver fluke infestations that is endemic to countries through which the Mekong River traverses on its route from Yunnan Province in China through to its delta in southern Vietnam.^6^

Liver fluke infection is caused by water-borne parasites, *Opisthorchis viverrini*, *Clonorchis sinensis*, and *Opisthorchis felineus*, which are transmitted to humans by the consumption of raw, pickled, or undercooked infected fish. *O. viverrini* is common in the Mekong Basin region, including Thailand, Laos, central Vietnam, and Cambodia, whereas in Korea, China, and northern Vietnam, *C. sinensis* is more prevalent. *O. felineus* is reported to be endemic in parts of Russia, Kazakhstan, and Ukraine.^7^

The distribution of biliary duct carcinoma in Thailand shows high variability (Figure 2); regions with high *O. viverrini* endemicity show higher CCA burden. In Southern Thailand, where *O. viverrini* infection is sparse, the incidence is 5.7 per 100,000, compared with 14.6 per 100,000 and 85 per 100,000 in the North and Northeast regions, where *O. viverrini* infects 19.3% and 15.7% of the population, respectively.^8^

### Liver flukes

Leiper reported the first human cases of *O. viverrini* infection in the cadavers of Thai prisoners in the early part of the last century.^9^ Eight decades later, in 1994, *O. viverrini* was classified, along with *C. sinensis*, as a group-1 carcinogen by the International Agency for Research on Cancer (IARC), based on their strong association with hepatobiliary carcinomas.^10^ The majority of opisthorchiasis and clonorchiasis cases are found in the Mekong River basin villages where the flukes infect around 90%–95% of cyprinid fish (fish with scales), and eating freshly caught fish raw is a long-standing tradition enjoyed among the locals.^11^ Koi pla (raw fish salad) and plasom (fermented fish) are a few of the most common infective dishes eaten in the region surrounding the 4,350-km long river, where fishing is one of the main sources of food and livelihood. Fish is the cheapest source of animal protein for the majority of the 67 million inhabitants of the Mekong Basin.^12^

The people of this region are also among the poorest in the world with an average income of <1 US dollar per day.^13^ Sithithaworn et al named the fluke infestation and its deadly consequences “the fatal disease of the poor”.^14^ Mortality from liver and bile duct cancer is the leading cause of death in Thai males and third in females, causing a total of 28,000 deaths per year. The highest number of liver cancer mortality cases is reported in the Northeast of Thailand, comprising 70% of the cases.^15^ *O. viverrini* is endemic in the wetland south of the Mekong region, affecting an estimated 8 million people in Thailand and 2 million in Laos. Until recently, the endemicity level in Cambodia was uncertain, but a large surveillance examining 16,082 fecal samples showed that the infection is highly endemic, reaching an alarming 65.1% in one of the villages in the Southern region. Eating raw fish in endemic areas was reported in 70%–100% of the locals.^16^ An estimated 601 million people are currently at risk of infection with *C. sinensis*, 570 million are in China and Taiwan alone, and 67.3 million people are at risk of *O. viverrini* infection.^7^

**Figure 3:**
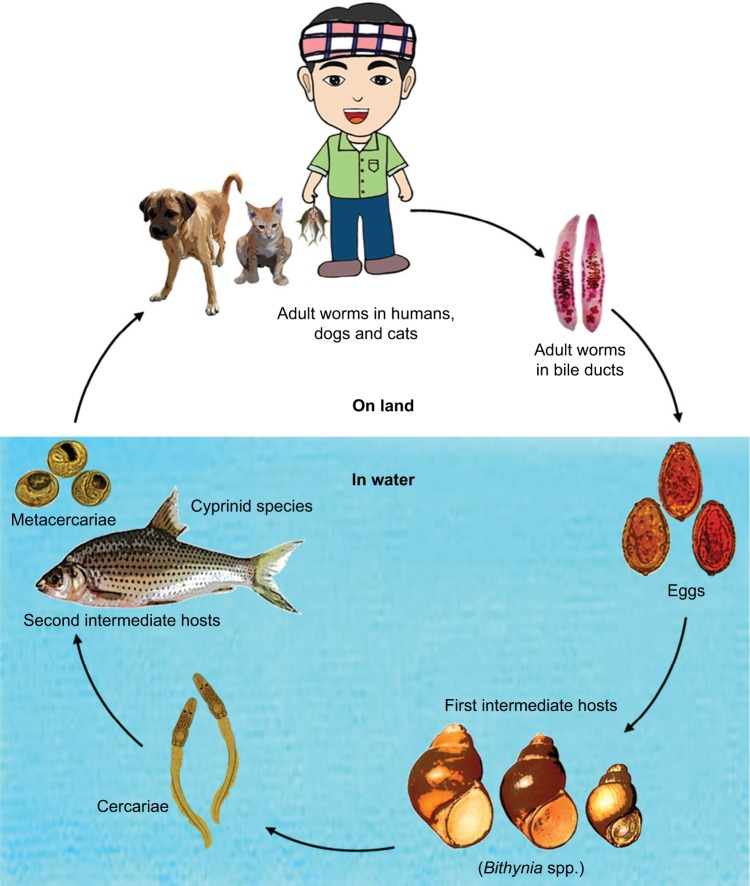
Liver fluke life cycle.

The fluke life cycle is complex, involving two intermediate hosts (snail to fish) and several morphological changes (Figure 3). After the ingestion of fish containing viable metacercariae, infected humans excrete the eggs produced by the mature adult worms in their feces. If they reach fresh water, the eggs are ingested by freshwater snails and the larva (or miracidia) hatch in the digestive tract of these snails. The infected snails shed thousands of cercariae into the water, which penetrate the skin between the scales of freshwater fish, and encyst, forming metacercariae. In 21 days they become infective with metacercariae, and when ingested by humans, they excyst in the duodenum and ascend to the bile duct via the ampulla of Vater. Using their two suckers and body contraction, the metacercariae migrate further into the smaller, proximal bile ducts where they become mature worms within a month and are able to sexually reproduce.

Adult worms (measuring 10–25 mm long and 3–5 mm wide) are able to produce 1000–5000 eggs per day, and are estimated to survive up to 25 years in the biliary tree, causing only mild symptoms.^10^,^17^ Conventional diagnosis of liver flukes is based on microscopic examination of the feces for eggs. The infection is usually asymptomatic in mild cases (fewer than 100 flukes). Malaise, abdominal discomfort, and diarrhea are sometimes present in early infection.^18^

Long-term complications associated with chronic infection include, hepatomegaly, cholecystitis, gallstones, and periportal fibrosis. The severity of such manifestations is linked to the intensity and duration of infection. Chronic inflammation is found to be a major etiological precursor of hepatobiliary malignancy, predominantly of CCA. Carcinomas typically present 30–40 years after infection, prognosis is generally poor, and death tends to occur within 3–6 months after diagnosis.^8^ Praziquantel, an anthelmintic drug, is used to eliminate the worms. Although it is an effective therapy, praziquantel does not reverse periductal fibrosis and inflammation in all patients; subsequently, it may not prevent the progression to CCA.^19^ In addition, the cycle of infection, dosing with praziquantel, and reinfection is recognized as a major challenge.^20^ Repeated infection cycles, typically by regular self-dosing with praziquantel after the initial infection to eliminate the worms, can potentially increase the susceptibility to cholangiocarcinogenesis due to oxidative stress and inflammatory responses.^20^

Chronic host inflammatory responses may persist even after the eradication of the liver flukes through the eggs they produce, which can remain entrapped in the periductal tissue causing granulomatous inflammation.^8^,^20^ Therefore, comprehensive assessment of reinfection rate and eradication potential is difficult to establish.

The association between the biliary parasitosis and CCA is well established, and extensively reviewed in the last two decades.^8^,^18^ CCA genesis is hypothesized to be initially induced via multifactorial mechanistic pathways: 1) mechanical damage caused by the fluke suckers, 2) fluke toxic secretary products, and 3) immunopathological host response. All of these factors result in chronic inflammation and hepatobiliary abnormalities, which trigger a proliferative response and formation of precursor lesions (eg, epithelial and adenomatous hyperplasia, and goblet cell metaplasia).^8^ Gene–environment interactions play an important role in the individual’s susceptibility to carcinogenesis. As an example, genetic polymorphism of glutathione-S-transferase, a detoxifying enzyme, is found to be associated with seropositivity for opisthorchiasis, and may in turn alter the individual’s risk to CCA.^21^

Nitrosamines, known carcinogens found in foods such as fermented food and commonly used fish paste in Northeastern Thailand and Laos, “plara”, add an additional risk factor.^8^ Some authors hypothesized that nitrosamine compounds are a primary carcinogen leading to hepatobiliarytumors.^18^

### Primary sclerosing cholangitis

PSC, a progressive cholestatic biliary disease with unknown etiology, is characterized by chronic inflammation that leads to destruction of the intra- and extrahepatic bile ducts.^22^ PSC incidence ranges from 0 to1.3 per 100,000 people/year in Western countries.^23^ The disease is asymptomatic in the majority of the patients, and is often diagnosed incidentally through routine check-up (eg, elevated liver enzymes). Fatigue is the most common symptom, whereas pruritus and jaundice occur in advanced stages as a result of cholestasis. Inflammatory bowel disease is highly common in PSC patients, up to 80%, and often diagnosed prior to hepatobiliary disease.^24^,^25^

The mean age at diagnosis with PSC is 40 years, with a median life expectancy of 9–12 years from diagnosis. PSC is associated with portal hypertension, cirrhosis, and hepatobiliary and colorectal cancer. Progression to CCA is unrelated to the duration of the inflammation.

It tends to be diagnosed in 6%–36% of PSC patients within a few years after diagnosis, making CCA from PSC onset at a younger age (∼34 years old), compared with the general population where biliary carcinoma commonly presents in the seventh decade of life.^25^ The only curative treatment to date for PSC is liver transplantation.^24^

### Biliary stones

Stones formed anywhere in the biliary tree are generally associated with increased CCA risk, substantially when the stones arise in the intrahepatic bile ducts, or hepatolithiasis, where it is estimated that 10% of the patients progress to develop iCCA. Hepatolithiasis rarely predisposes to CCA in Western patients, but it is a relatively common risk factor in several Asian countries, where the incidence ranges from 2% to 25%.^26^ The presence of intrahepatic biliary stones was found in nearly 70% of CCA patients undergoing surgical resection in Taiwan.^27^

Hepatolithiasis is typically concomitant with biliary stasis, cholangitis, strictures, and bacterial infections, subsequently, leading to prolonged inflammatory status and biliary injury, which in turn triggers malignant cholangiocyte growth, in some cases.^26^ Cholecystolithiasis (gallbladder stones) and choledocholithiasis (stones in the common bile ducts) were found to be associated with increased intrahepatic cholangiocarcinogenesis with an odds ratio of 4.0 and 23.97, respectively, in a large-scale Danish case-control study (n=764 iCCA cases and n=3,056 population controls).^28^

### Choledochal cysts

Choledochal cysts are a rare congenital dilatation of the biliary tree (incidence of 1 per 100,000 people). The cysts can be single or multiple, and arise in the intra- or extrahepatic bile ducts. The disease is idiopathic, and is mostly common in Asian females. Several hepatobiliary complications can present with choledochal cysts including, cholelithiasis, cholecystitis, cholangitis, cirrhosis, portal hypertension, pancreatitis, pancreatic duct obstruction, and CCA. The risk of CCA in patients with choledochal cyst is 10%–30%, and even though surgical resection of cysts reduces the risk to 0.7%–6%, malignant transformation can still occur, and life-long follow-up is recommended for the majority of patients.^25^,^29^ Moreover, the coincidence of abnormal pancreaticobiliary duct junctions increases the possibility of cholangiocarcinogenesis. Pancreatic enzymes reflux, cholestasis, and elevated bile acid concentrations can further induce malignant proliferation in patients with choledochal cysts.^29^

### Chemical carcinogens

Exposure to Thorotrast, a radiographic contrast agent banned in the1960s, is reported to be associated with a 300-fold increased risk of CCA, occurring 16–45 years after exposure.^30^ Thorotrast is primarily deposited in the liver (∼60%), and continues a lifelong emission of internal radioactive particles, as it has a biological half-life of 400 years.^31^ Pathogenesis from Thorotrast exposure to carcinogenesis has not been fully proven. Radiation-induced liver insult has been shown to promote hepatocyte remodeling, genomic instability, DNA methylation, and disrupt the liver architecture. Few genetic mutations have been reported to be associated with Thorotrast-induced hepatobiliary malignancy, such as K-*ras*-2 and *TP53*.^32^

In addition, occupational exposure to a halogenated hydrocarbon solvent, 1,2-dichloropropane, was found to be associated with CCA among printing factory workers in Japan, where the substance is used in ink removal. Long-term exposure (via inhalation and dermal contact) was linked to CCA induction among the young factory employees. The substance was categorized as class-1 carcinogen in 2014 by the IARC.^33^

### Other risk factors

Other hepatic diseases associated with CCA include alcoholic liver disease, cirrhosis, and cholangitis with odds ratios of 19.22, 75.9, and 6.3, respectively, reported in a Danish cohort.^28^ CCA risk in viral hepatitis was recently investigated in a Korean study with n=276 CCA cases, hepatitis B virus significantly increasing iCCA risk with an odds ratio of 4.1.

The risk is greater in the presence of diabetes (odds ratio=12.2), and diabetes independently was associated with both intra- and extrahepatic biliary carcinoma.^34^ Obesity was unrelated to CCA risk in both the Korean and Danish cohorts.

Inflammatory bowel disease is also implicated in cholangiocarcinogenesis. This, however, might be confounded by the presence of PSC which is often not controlled for in epidemiological studies. Data on alcohol intake and smoking in association to CCA risk are inconsistent, but they are both recognized as potential risk factors.^25^ Several genetic polymorphisms related to cellular DNA repair, protection against toxins, or immunological surveillance have been implicated in biliary tract carcinogenesis.^2^

## Tumor phenotypes

**Figure 4:**
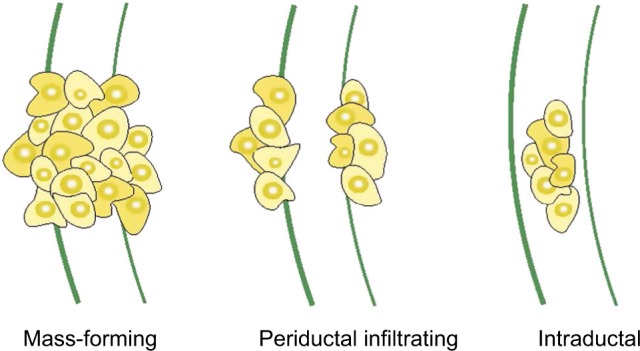
Morphologic classification of CCA. **Note:** The gross appearance of CCA tumors can present with three patterns of growth: mass-forming, periductal infiltrating, and intraductal. **Abbreviation:** CCA, cholangiocarcinoma.

CCA is an extremely heterogeneous group of malignancies in its genomic, histological, morphological, biological, and clinical features. Apart from the anatomical classification of tumors (ie, intrahepatic, perihilar, or distal), they are further divided into mass-forming, periductal, and intra-ductal, based on their gross appearance, as illustrated in Figure 4. CCAs can present as a single or multiple growth type, such as periductal infiltrating plus mass-forming. The most common growth type is mass-forming, accounting for 40%–42% of tumors.^35^

Histologically, >90% of CCA are adenocarcinomas. Other microscopic types include adenosquamous and squamous carcinoma, and rare subtypes.^36^ The tumors show various grades of differentiation: poor, moderate, and well. Those with low-grade or well-differentiated types have better prognosis and lower incidence of distal metastases than with high-grade carcinomas.^35^ Combined hepatocellular–CCAs can present within the same liver. It is a rare type of primary liver cancer that shows histopathological features of both HCC and CCA, and accounts for 1%–15% of all CCAs.^36^

Various risk factors of CCA and associated pathologies, diverse histological features, and tumoral genomic heterogeneity cast doubt on the cellular origin. Additionally, due to the fact that CCA can arise as a result of hepatocyte injury, such as viral hepatitis and alcoholic liver disease, it is probable that CCA can arise from hepatocytes, and not invariably from the cholangiocytes lining the biliary tree.^37^

CCA carcinogenesis from hepatocytes was observed incidentally via the activation of the oncogenes, NOTCH and AKT, in vivo. Evidence from hepatocyte fate-tracing experiments, which enables tracking of the fate of cells in living mice, showed that activated NOTCH signaling, in combina tion with AKT overexpression, promotes tumorigenesis in the early stages of intrahepatic bile duct carcinomas.^38^ NOTCH signaling is also known to play an important role during the embryonic development of liver architecture, including cholangiocyte differentiation and biliary duct development.

Genome-wide analysis studies revealed the global gene expression patterns and transcription mutations in CCA. Intrahepatic tumors showed greater genomic heterogeneity than tumors arising outside the liver (2,354 genes with altered expression vs 545).^39^ To further capture CCA molecular profile, somatic mutational screening studies identified several mutated genes. *KRAS* mutations and loss-of-function mutations of *TP53* are the most commonly altered genes in CCA, reported in around 10%–45% and 21% of cases, respectively. Multiple gene alterations were identified by various molecular profiling techniques and described molecular heterogeneity of CCA tumors. Different molecular expression profiles were associated with the tumor anatomical location, and several mutations were shown to be significantly associated with poor prognosis. The molecular mutations that characterize CCAs include *IDH1*, *IDH2*, *ARID1A*, *PIK3CA*, *BAP1*, and *NRAS*.^40^

Furthermore, exome sequencing has identified characteristic mutational patterns in liver fluke-related bile duct cancers, distinct from non-infection-related bile duct cancers.^41^ In addition, it is of interest to note that infection with *O. felineus* induces intraepithelial neoplasia of the biliary tract in a rodent model, further garnering evidence for the direct link between liver-fluke infections and CCA.^42^

Overall, tumoral phenotypic characterization (genomic, epigenetic, and molecular) is associated with tumor aggressiveness, prognosis, and response to therapy, thus potentially indicating the best therapeutic approach to tailor personalized treatment for each patient. Therefore, it should be a key strand of future research to identify new potential therapeutic targets for treating CCAs, based on their phenotypic signature, in order to enable patient stratification.^3^

## Diagnosis

CCA diagnosis is often complex and requires the use of multiple diagnostic modalities to 1) establish strictures anatomical location; 2) distinguish between benign and malignant strictures; 3) differentiate CCA from gallbladder, other primary liver tumors, and combined hepatocellular–CCAs; 4) stage and grade the tumors; and 5) plan treatment approach. This is often difficult owing to the tumor anatomical location, desmoplastic nature, and lack of definitive diagnostic tests.^36^,^43^

### Clinical presentation

Clinical symptoms and signs of CCA are often dependent on where the lesions originate in the biliary tree and are typically absent in early stage tumors. Malignant strictures originating within the right or left intrahepatic bile ducts tend to cause nonspecific symptoms (such as weight loss, abdominal discomfort, and malaise). Jaundice, pruritus, and pale stool are associated with lesions originating from the hilar and distal biliary ducts when the tumor is large enough to obstruct the biliary flow.^36^

### Blood biomarkers

To date, there is no specific blood test available to diagnose CCA. Conventional liver function markers such as serum bilirubin, alkaline phosphatase, and aminotransferase enzymes are elevated in the presence of biliary obstruction, and are not indicative of malignancy per se.^43^

Serum tumor markers, carbohydrate antigen (CA)19–9, CA-125, and carcinoembryonic antigen (CEA) are the most widely used markers for suspected CCA. They are clinically used in conjunction with other diagnostic tools, and should not be used alone due to their poor diagnostic performance and inherent limitations.^36^

For example, CA19–9, the most used marker, is nonspecific to CCA and is elevated in pancreatic, colorectal, and gastric cancers, and also in nonmalignant conditions, such as PSC and obstructive jaundice. In a follow-up study, 37% of PSC patients with elevated CA19–9 were negative for CCA.^44^ Patients lacking Lewis antigen (10% of the general population) cannot produce CA19–9 and will not benefit from CA19–9 testing. The sensitivity and specificity of CA19–9 in CCA patients range from 40% to 70% and 50% to 80%, respectively, with a positive predictive value of 16%–40%.^36^

CEA is raised in 30% of patients with CCA, whereas CA-125 is elevated in around 40%–50%. Both markers are nonspecific and insufficient for accurate diagnosis.^36^ Several other potential serum tumor markers have been linked to CCA (eg, CYFRA21–1, CA-195, CA-242, DU-PAN-2, IL-6, and trypsinogen-2), but their clinical utility is still unclear, and they are not in routine use.^36^,^45^

### Imaging and other techniques

In suspected CCA, ultrasonography (US) is initially used to exclude gallstones, assess biliary dilatation, and localize the obstruction site. Yet, US alone is insufficient to confirm diagnosis; it is operator-dependent, cannot accurately define tumor extent, and may miss small tumors. High-resolution imaging, computed tomography, and magnetic resonance imaging (MRI) provide greater sensitivity for CCA detection than US, but may not be readily accessible in Thailand and resources-limited settings. MRI with cholangiopancreatography is the technique of choice to visualize the ductal and vascular structure, define tumor extent, and detect distant metastases. The advantages of the procedure is its high sensitivity and specificity, noninvasiveness, and the fact that there is no risk of radiation.^43^

Endoscopic retrograde cholangiopancreatography (ERCP) and percutaneous transhepatic cholangiography are more invasive, but they offer the possibility of therapeutic intervention, as well as diagnostic assessment. They allow sampling of strictures for brush cytology or tissue biopsy, and stent insertion to relieve biliary obstruction. These sampling methods are highly specific (100%), but they have a low sensitivity(46%–73%). Cytology is positive in <50% of CCA cases. Thus, negative results do not exclude malignancy. Biopsy, in addition to cytology, can enhance positive findings to 40%–70%, but tissue is not always easy to obtain, owing to the scirrhous nature of desmoplastic tumors and risk of tumor seeding.^36^ Once CCA is confirmed, comprehensive staging must be carried out to screen for metastatic disease. The above diagnostic techniques are complementary and selection is dependent on the clinical needs of the individual. Multiple imaging techniques might be necessary for surgical assessment of resectability.

### Treatment and prognosis

Surgical resection is the only curative therapy for CCA. Patients with intrahepatic tumors are candidates for partial hepatectomy, whereas pancreatoduodenectomy is most suitable for distal tumors. For pCCA, the extent of surgical intervention required is based on Bismuth–Corlette classification, and it is generally associated with the worst resectability rate, compared with distal tumors (56% vs 91%).^46^

The majority of CCA cases present too late for surgical intervention. The latest treatment guidelines recommend a combination of Gemcitabine and Cisplatin chemotherapy for inoperable cases. This approach was adapted following encouraging results from two large randomized studies, ABC-02 in the UK and BT22 in Japan. The regimen is an acceptable standard first-line therapy for patients with advanced biliary tract cancer, where in some cases it was successful to downstage tumors to operable state and improve survival.^36^,^47^

Currently, there is no evidence to support the routine use of radiotherapy postoperatively or for unresectable disease.^3^,^36^

Srinagarind Hospital, the leading center in CCA treatment in the Northeast of Thailand, published a prospective study on Thai CCA patient outcome. Out of 163 patients with biliary carcinoma, only 10 cases (6.1%) were eligible for curative resection. The vast majority presented too late for any intervention, stage IV, and were referred to palliative care from the first visit. The overall median survival was 4 months, but patients who received surgical resection with negative tumor margins had 100% 4-year survival rates.^48^

The prognosis for inoperable Thai patients with bile duct cancer receiving palliative chemotherapy is poor. Gemcitabine chemotherapy has a median survival of 10 months.^49^ Moreover, resection margins are important prognostic factors, and complete surgical resection at an early stage is the only treatment that significantly improves patient survival.

Long-term survival and recurrence rate post hepatic resection were examined in a Korean retrospective study: iCCA recurred in 60% of cases, either in the remnant liver and in lymph nodes or as distant metastasis, commonly in the lung. Tumor size, lymph node metastasis, and multiple tumors were significant predictors of recurrence.^50^

Molecular therapies for solid biliary tumors are generally complex to develop, owing to their therapeutic resistance, and anatomical, histological, and phenotypic heterogeneity.^2^ Elucidation of the molecular mechanisms and pathways underlying cholangiocarcinogenesis may expedite development of personalized therapy for patients based on their particular tumor phenotype.

## Screening strategies

Screening strategies for CCA vary according to the disease etiology, where it moves from small-scale, high-risk patient surveillance, such as for PSC patients, to large, community-based screening programs as in the *O. viverrini* prevention campaign in Thailand.

### Cholangiocarcinoma screening in Western countries

Established risk factors account for less than a third of CCA cases in the West.^36^ Due to the disease rarity and the ambiguous definition of asymptomatic at-risk population, surveillance guidelines of sporadic CCA are yet to be established. Patients with predisposing hepatobiliary disease undergo series of annual screening tests. PSC patients are typically screened mainly for CCA, but also for HCC and colorectal cancer. If biliary dominant strictures are present, regular ERCP with brush cytology is recommended. This is not ideal for surveillance; it is invasive, costly, and has low diagnostic accuracy.^51^ Solely screening for CCA might not be feasible. To overcome this limitation, it is proposed to design a multicancer detection panel that combines CCA markers with other markers of uncommon respiratory and digestive tract cancers or aerodigestive cancers. This screening modality may justify and permit CCA screening in countries with low incidence.^51^

### Cholangiocarcinoma screening and control in Thailand

Approximately, 36% of the Thai population (23 million) live around the Mekong river wetlands, and are at potential risk of opisthorchiasis.^52^ Primary prevention of CCA through opisthorchiasis control began in Thailand in 1950 as local treatment units in few high-endemic provinces. When praziquantel was introduced in the early1980s, the Thai Ministry of Health started four opisthorchiasis treatment units, which examined over half a million people and treated 400,452 cases.^53^

In 1987, a national-scale opisthorchiasis screening program began as a part of the country’s national health plan. Over 5 million stool samples were examined, and 1.8 million positive cases were identified and treated in the North-Eastern region. The program expanded to cover provinces in the North and Central Thailand in 1992, resulting in a fall in the national opisthorchiasis prevalence, from 63.6% to 9.6% in 2001.^53^

Despite the intensive health campaigns targeting *O. viverrini* prevention, the prevalence remains remarkably high in some parts in Thailand, and subsequently, CCA burden remains alarmingly high. Opisthorchiasis prevalence reached 42.2% in the latest epidemiological surveillance, named the Cholangiocarcinoma Screening and Care Program (CASCAP). CASCAP was developed by the Medical Faculty of Khon Kaen University in cooperation with the Cholangiocarcinoma Foundation, as a community-level screening program that aimed to document the current status of opisthorchiasis-related CCA in the region.^54^

Repeated infection is likely to occur after the administration of praziquantel and might explain the persistent high infection rate. In an animal model, repeated infection of hamsters with *O. viverrini* exhibited an inflammatory response and showed higher levels of parasite-specific antibody, which positively correlated with a greater degree of fibrosis.^55^ About 89% of the CASCAP cohort had previously eaten raw or fermented fish,^54^ indicating a poor response to the educational campaigns against raw fish consumption among rural communities. Moreover, the vast majority are farmers (79.9%), and raw fish based-diet is embedded in their cultural identity. The magnitude of the problem is greater than telling people to “stop eating raw fish”; it has social, political, and environmental implications.

Additionally, the perceived political tension between the rural Northeast and the Bangkok government may have created resistance in accepting the health promotion messages communicated from the government to villages.^56^ Reinfection cycles are also influenced by poor sanitation practice and lack of sewerage infrastructure. Commonly, during important growing/harvest cycles in the rice-fish Mekong Basin cultures, temporary homes are used which are typically huts with no latrines.^53^

Currently, the screening modality used in Thailand is US imaging-based, which has several limitations. Experienced radiologists are required, and the effectiveness of the diagnosis depends on the operator skills. US is time-consuming, and not suitable for rapid screening of at-risk people in endemic areas. The rate of early tumor detection using US is unknown, and repeated US procedure is required for at-risk patients every 6–12 months; therefore, storage of the digital information require reliable server and network infrastructure. On the other hand, US is noninvasive, and with extensive training, optimizing sonographic equipment, and procedure standardization, US was shown to have early CCA detection potential by the detection of periductal fibrosis.^57^

The absence of prognostic techniques limits therapeutic options. Therefore, there is an urgent unmet demand for the development of biomarkers that hold clinical potential for timely diagnosis of the CCA in Thailand and neighboring countries. CCA incidence in Thailand appears to be increasing, not decreasing, and even if the worm is eradicated, it will take 20–30 years to achieve CCA-free population. Hence, those with history of parasitosis are still at risk of developing malignant biliary tumors.

## Future directions

The mortality from CCA remains equal to the disease incidence. New biomarkers for screening, diagnosis, staging, and prognosis would be crucial in the management of CCA, as current methods are ineffective in detecting small tumors. To date, several serum markers, including the clinically available iCA19–9 and CEA, and potential markers such as CA242, mucin glycoproteins, and several cytokines have been reported to be adequate diagnostic and prognostic markers for CCA.^58^ Yet, the diagnostic accuracy of such markers is not sufficient for a timely and reliable diagnosis of CCA.

A high-throughput “omics” approach and the development of multi-marker panel would be of future benefit to explore potential diagnostic targets in disease screening applications. At the individual level, metabolic profiling provides the possibility of personalized health care, which aims to deliver targeted therapies and predict individual’s response to treatment.^59^

Such methodology may have the potential to provide a new diagnostic window to CCA. Furthermore, metabolic phenotyping of CCA may shed light on understanding environmental interactions underpinning CCA genesis and may lead to the discovery of diagnostic markers in biofluids.
